# Protocol for extracting and evaluating activity distributions in rat electrocorticograms with principal component analysis and k-nearest neighbor

**DOI:** 10.1016/j.xpro.2025.104041

**Published:** 2025-08-18

**Authors:** Astrid Mellbin, Udaya Rongala, Fredrik Bengtsson

**Affiliations:** 1 Neural Basis of Sensorimotor Control, Department of Experimental Medical Science, Biomedical Centre, Lund University, SE-223 62 Lund, Sweden

**Keywords:** Model Organisms, Neuroscience, Systems biology

## Abstract

Principal component analysis (PCA) and k-nearest neighbor (kNN) can be applied to extract and compare activity distributions from electrocorticogram (ECoG) signals across recorded neural activity. Here, we present a protocol for recording ECoG activity from the neocortex of rats and applying PCA and kNN on the recorded data. This protocol allows for comparison between different types of cortical activity in a multidimensional space.

For complete details on the use and execution of this protocol, please refer to Mellbin et al.^1^

## Highlights

• Steps for setting up electrical equipment to record ECoG activity
• Procedures for craniotomy and placement of electrodes
• Instructions for recording and processing the ECoG data
• Guidance on performing PCA and kNN to analyze ECoG data

## Abstract (editor highlights)

Publisher’s note: Undertaking any experimental protocol requires adherence to local institutional guidelines for laboratory safety and ethics.

## Graphical abstract

**Figure:**
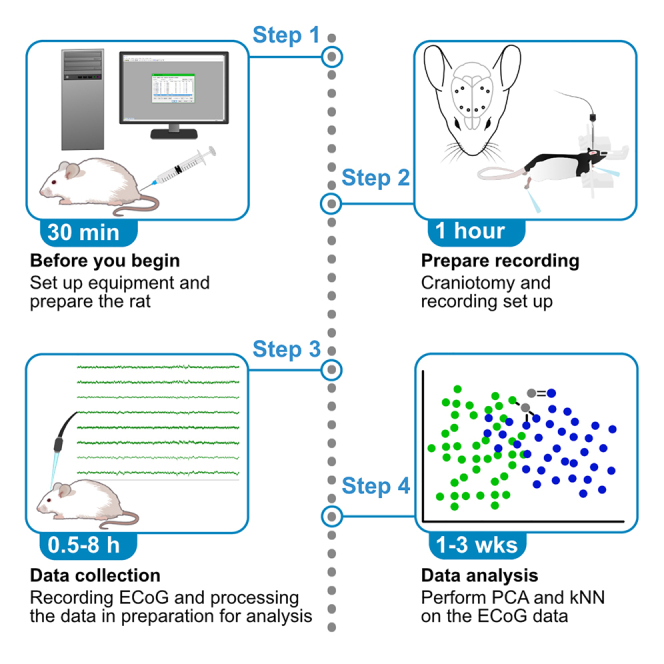

## Before you begin

As growing evidence supports the existence of globally interconnected functional networks within the neocortex, there is an increasing demand for tools capable of analyzing the activity of multiple brain regions simultaneously and assessing their interrelationships.^2^^,^^3^^,^^4^ While several such methods exist, each presents certain limitations. For instance, calcium imaging provides excellent spatial resolution but suffers from limited temporal resolution, whereas traditional EEG spectrogram analysis lacks the ability to assess interregional (cross-channel) dynamics.^2^^,^^5^

**Figure 1:**
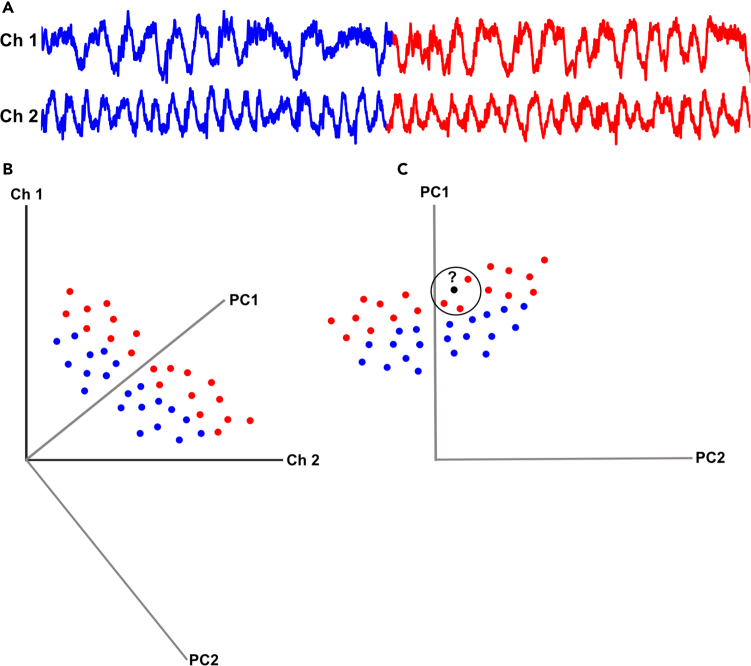
Overview of the basic principles of the analytic methods (A) Example of data recorded from two ECoG channels. The different colors represent two different types of fictional activity. (B) The positioning of the data at each time step in a two dimensional space based on the value in each of the channels. Colors indicate what part of the recorded trace it belongs to. In order to make the example as clear as possible, the coordinates of each point is fictional. The gray vectors show where the principal component analysis might create new vectors to capture as much of the information as possible. (C) How the data from (B) would be positioned in the new coordinate system. Please note that while the positioning from both the Ch1 and Ch2 vector would be needed to know if a dot was red or blue in (B), here it can be almost completely separated only by its positioning along the PC1 vector. The kNN analysis will then attempt to classify an individual dot simply by examining the class of a defined number of dots closest to it. In this example the number of neighbors is set to three.

This protocol outlines a method for acquiring ECoG data from rats and analyzing it using PCA and kNN classification. This approach offers a high temporal resolution and can be used to assess the activity distribution of neural activity across cortical regions and how these patterns relate to one another (Figure 1).

### Institutional permissions

All animal procedures were done according with institutional guidelines and were approved ahead of time by the Local Ethics Committee of Lund, Sweden.

Researchers using this protocol must have permission and follow the rules of their local authorities.

### Setup: Recording and stimulation equipment and hardware

**Timing: 30 min**

**Figure 2:**
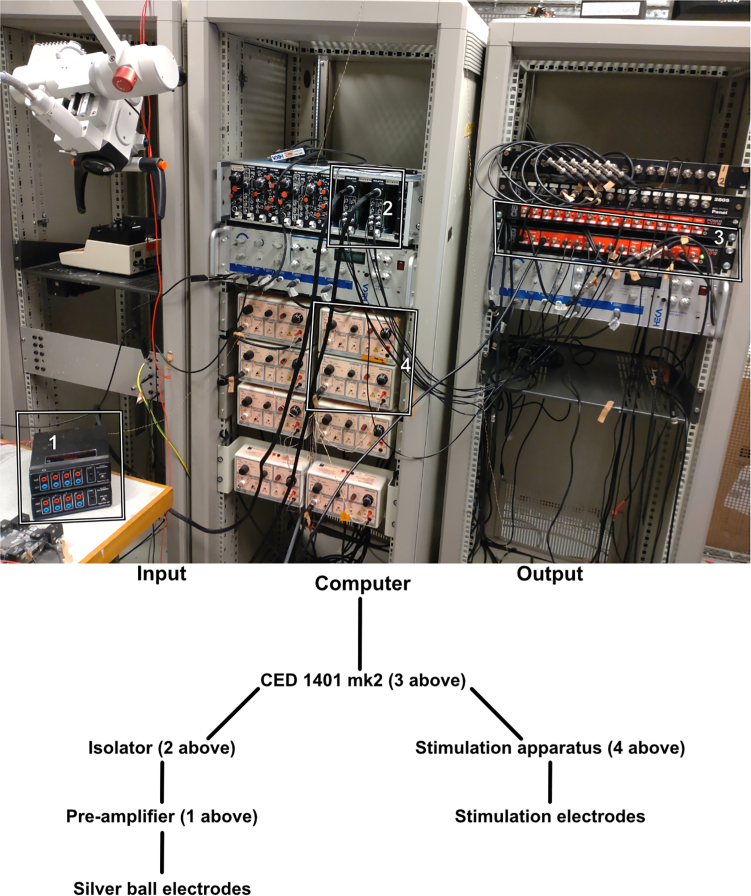
Set up for recording input and output On top is shown the set up from the lab, below is a schematic of the set up. 1. Pre-amplifier Digitimer NL844. 2. Isolator Digitimer NL820. 3. CED 1401 mk2. 4. Stimulation apparatus Digitimer DS3.

This step-by-step guide describes how to set up the recording and stimulation equipment, including the configuration of relevant parameters (see Figure 2).

1. Prepare the pre-amplifiers and isolator.
  a. Connect the pre-amplifiers to the isolator.
    **CRITICAL:** Make sure to note how the pre-amplifiers are connected to the isolator to ensure that the correct ECoG trace is attributed to each cortical area.
    ***Note:*** Here the pre-amplifier used is of the type “Digitimer NL844” and the isolator is a Digitimer NL820A isolator (Neurolog system, Digitimer). Other pre-amplifiers and isolators of similar ability can be used.
  b. Set the pre-amplifiers low frequency cut off at 0.1 Hz and gain at ×1000.
  c. Set the isolator to gain x5.
  d. Connect the isolator to the CED 1401 mk2.
2. Prepare the stimulation electrodes.
  a. Connect the CED 1401 mk2 outputs to the stimulation apparatus (Digitimer DS3).
  b. Connect stimulation electrodes to the output outlet.
  c. Set the output amplitude to your desired amplitude on the DS3.

***Note:*** In our paper a pulse intensity of 0.5 mA was used.

**CRITICAL:** Make sure that the stimulation electrodes are connected to the ports corresponding to the correct stimulation output.

3. Prepare your recording system.
  a. Connect CED 1401 mk2 to your computer.
  b. In the spike2 software, create a configuration with the desired number of waveforms and events for input and output.
    ***Note:*** Handbooks for the CED 1401 and spike2 software can be found online (https://ced.co.uk/products/spkovin).
  c. Create your desired output sequence with your desired pulse duration and interval.
    ***Note:*** In our paper a pulse duration of 0.14 ms was used and a pulse interval corresponding to a stimulation frequency of 0.3–5 Hz.
  d. If using MATLAB for the analysis, download the smr to mat.s2s code from GitHub (https://github.com/Neural-basis-of-sensorimotor-control/matlab-analysis/blob/master/smr%20to%20mat.s2s).
    ***Note:*** Other recording systems that allow for recording multiple simultaneous waveforms as well as outputs can be used. The minimum hardware requirements for the latest version of spike2 is Windows 10 with 86× or 64× CPU and 4 GB of memory.

### Preparation and surgery

**Timing: 1–3 h**

This step covers the surgery and anesthetic procedures.

**Figure 3:**
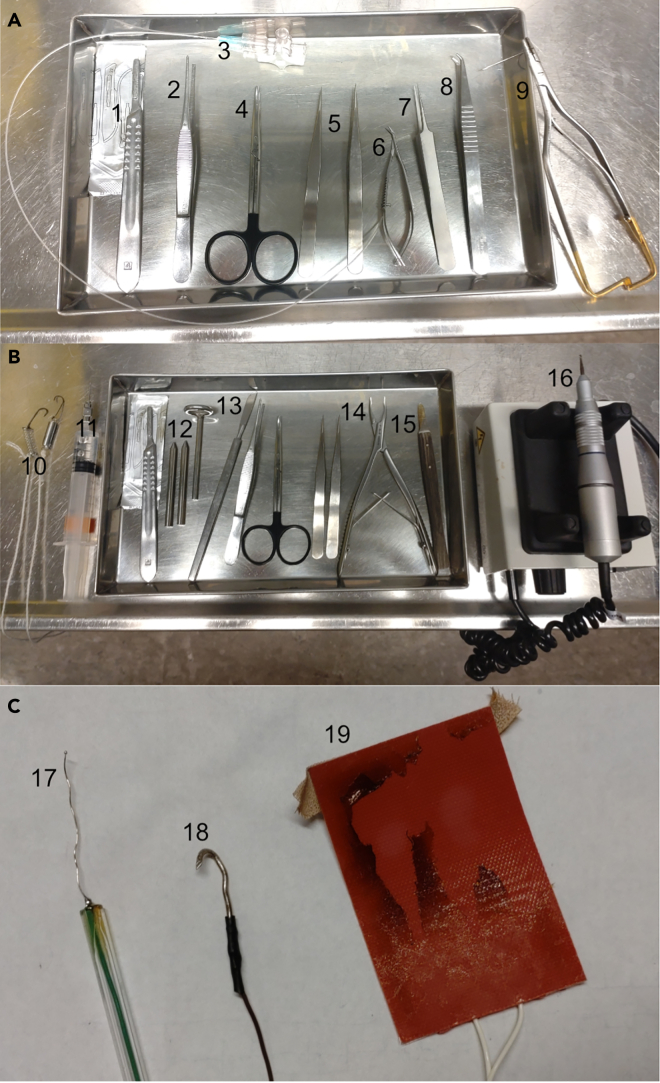
Surgical instruments used (A) Instruments used for preparatory surgery. 1. Scalpel (sharp). 2. Tissue forceps. 3. Vein catheter. 4. Dissection scissors. 5. Surgical fine forceps. 6. Vascular scissors. 7. Microsurgical forceps (tip size 0.005 × 0.25 mm). 8. Catheter tweezers. 9. Forceps and surgical needle. (B) Instruments used for craniotomy. 10. Wound retracting hooks. 11. NaCl. 12. Stabilizing ear rods and snout stabilizing ring. 13. Fixed scalpel (blunt). 14. Bone Rongeur forceps. 15. Bone wax scalpel. 16. Dental drill. (C) Electrodes and heating pad. 17. Silver ball electrode Φ250 μm. 18. Grounding electrode. 19. Heating pad, removed from the steel frame.

4. Prepare for the surgery.
  a. Gather the necessary tools and instruments (Figure 3A).
  b. Make sure the hair clipper is charged.
5. Prepare two separate batches of ketamine/xylazine mixture.
  a. Make one batch with a ketamine: xylazine concentration ratio of 15:1 for the intraperitoneal injection.
  b. Make one batch with a ketamine: xylazine concentration ratio of 20:1 for the continuous intravenous infusion.

**CRITICAL:** Clearly mark which batch is which to ensure the correct batch is used.

**Figure 4:**
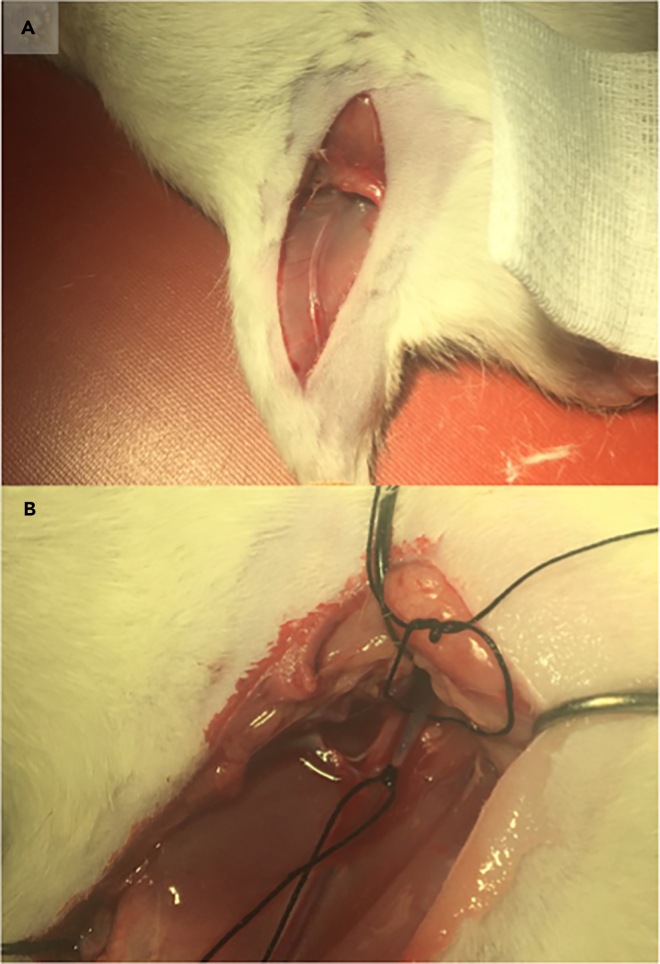
View of the femoral vein during preparatory surgery (A) The vein being just visible after the first dissection. (B) The placements of the surgical sutures to cut off the venous blood flow and lock the catheter in place.

6. Weigh the rat so that a correct inducing dose of anesthesia (IP: 0.0014 mL/g, IV 0.0006 mL/h/g) can be administered.
7. Anesthetize the rat.
  a. Put the rat in a container with controlled ventilation.
  b. Cover the container with a dark cloth to give the rat a calm environment.
  c. Start a flow of isoflurane gas and oxygen mix to the container (3% isoflurane).
  d. Leave the rat for 1–2 min, until deeply sedated.
    **CRITICAL:** Ensure that the container has a controlled inflow and outflow.
  e. Move the rat to the operation table and inject the ketamine/xylazine mixture intraperitoneally for a more long-lasting anesthesia.
  f. Ensure that the level of anesthesia is deep enough by making sure the withdrawal reflexes in response to a noxious pinch to the hind paw is absent.
8. Insert the intravenous catheter into the femoral artery (Figure 4).
  a. Put the rat on its back.
  b. Stretch the hindleg and restrain it.
  c. Shave the area above the femoral vein.
  d. Using the scalpel, cut through the skin above the femoral vein.
  e. Remove the connective tissue above the femoral vein using blunt dissection.
  f. Once the femoral vein and artery are visible, continue with careful blunt dissection using tweezers to free and separate the vein from the artery (Figure 4A).
  g. Once the femoral vein is freed, use surgical fine forceps to pull off any connective tissue on the femoral vein.
    **CRITICAL:** Be very careful when removing the connective tissue on the femoral artery, to avoid damaging the vein. Troubleshooting 1.
    ***Note:*** Take care to remove as much connective tissue as possible, as otherwise the catheter might accidentally be inserted between the connective tissue and the vein, rather than in the vein.
  h. When all connective tissue is removed, prepare by putting two surgical sutures under the vein, one below the planned insertion site, and one above.
  i. Tie the suture below the planned insertion site to cut off the blood (Figure 4B).
  j. Using vascular scissors, cut a small hole in the femoral vein.
    **CRITICAL:** Make sure to be careful when cutting the vein. An opening too small risks it being impossible to insert the catheter. Too big and the vein might be completely cut through and/or the rat bleeding out.
  k. Use microsurgical forceps (tip size 0.005 × 0.25 mm) to gently lift the upper edge of the vein incision and insert the catheter past the upper ligature.
  l. Aspirate for blood to ensure that the catheter is placed correctly and tie the upper ligature tight around the vein and the catheter to ensure that the catheter stays in place.
  m. Administer a first dose of intravenous anesthesia and check for leakage.
9. Finish the surgery.
  a. Put a small piece of cotton soaked in 0.9% NaCl solution in the surgical wound to keep the tissue moist.
  b. Close the wound using surgical suture.
  c. Carefully move the rat to the stereotaxic frame and attach the femoral catheter to the intravenous infusion apparatus.
  d. Start the anesthetic infusion and adjust the infusion rate according to the rat’s body weight to maintain an appropriate level of anesthesia.

**CRITICAL:** Do not pull on the femoral catheter while moving the rat or it might fall out.

***Note:*** Depending on how long the preparatory surgery takes, additional anesthesia might have to be administered intraperitoneally, it is important to keep monitoring the depth of anesthesia.

### Preparation for craniotomy

**Timing: 10 min**

This step details the final preparations for the craniotomy.

**Figure 5:**
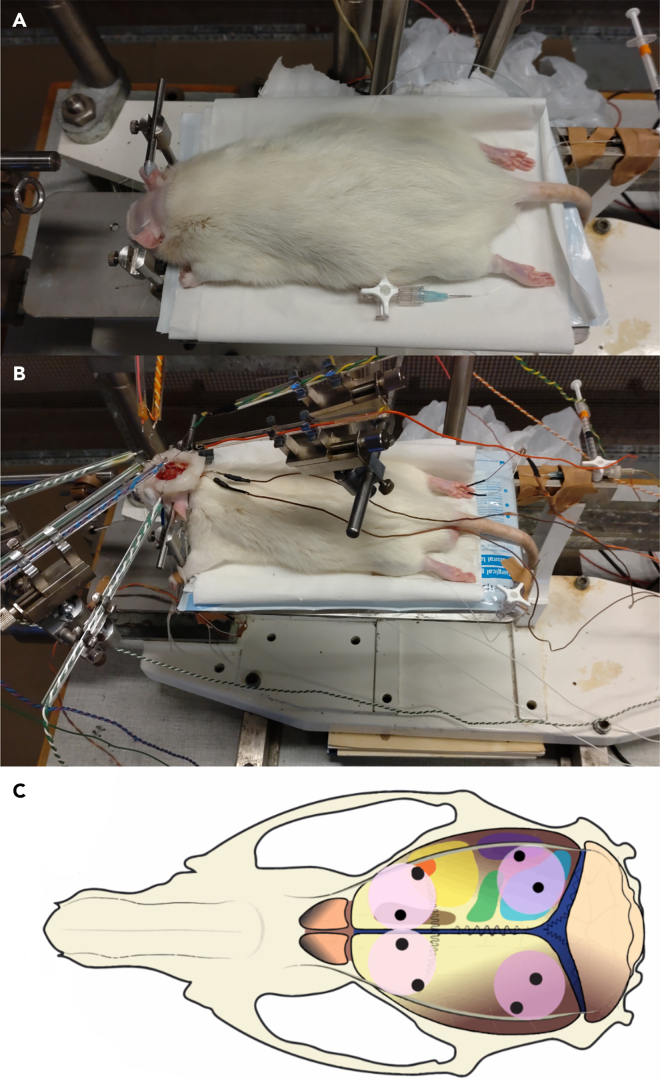
View of the rat during craniotomy and recording (A) Showing the shaved area of the head, and the placing of the first ear stabilizing rod, before the rat is mounted and fixated in the stereotaxic frame. (B) Showing the complete recording set up, with the head fixation, agar pool, recording electrodes and stimulation electrodes. (C) Schematic drawing of the craniotomy placement and placement of the recording electrodes on the rat’s skull and brain.

10. Prepare the tools for craniotomy.
  a. Gather the necessary tools and instruments (Figure 3B).
  b. Mix agar-agar with NaCl solution and put on a magnetic stirrer with a hot plate to mix and heat.
  c. Cut out small (approx. 1 × 3 mm) strips of Spongostan.
  d. Put bone wax on a warm surface to soften.
  e. Put paraffin oil in a warm water bath to heat to 38°C.
11. Fixate the rats head.
  a. Put xylocaine salve on the ends of the stabilizing ear rods and insert into the rat’s ears and fixate in the stereotaxic frame (Figure 5A).
    **CRITICAL:** Make sure the stabilizing ear rods are properly inserted in the external acoustic meatus to minimize discomfort.
  b. Put the snout stabilizing ring on the rat’s upper jaw. Make sure the front teeth are through.
  c. Move the tongue to the side of the mouth to ensure no pressure is put on it.
  d. Shave of the fur on the scalp.

## Key resources table

**Table:** 

REAGENT or RESOURCE	SOURCE	IDENTIFIER
**Chemicals, peptides, and recombinant proteins**		

Saline solution 0.9%	Sigma-Aldrich	S8776
Ketamine (Ketaminol vet 100 mg/mL)	Apoteket	EAN: 7046265115199
Xylazine (Rompun vet 20 mg/mL)	Apoteket	EAN: 7046260235724
Ringer-acetate	Apoteket	EAN: 55413760415012
Glucose 50 mg/mL buffered	Apoteket	EAN: 7046261310376
Xylocain (10 mg/mL)	Apoteket	EAN: 5060249175421
Agar	VWR	A10752.36
Paraffin oil	VWR	1.07160.1000
Isoflurane (IsoFlo vet)	Apoteket	EAN: 5702670021853
Pentobarbital (Euthasol vet. 400 mg/mL)	Apoteket	EAN: 8719874711159

**Experimental models: Organisms/strains**		

Rat: RjHan:SD male, 65–105 days old	Janvier Labs	https://janvier-labs.com/en/fiche_produit/sprague_dawley_rat/

**Software and algorithms**		

MATLAB	MathWorks	RRID:SCR_001622 https://se.mathworks.com/products/matlab.html
Spike2 v.7	Cambridge Electronic Design (CED)	RRID:SCR_000903
smr to mat.s2s	Hannes Mogensen, GitHub	https://github.com/Neural-basis-of-sensorimotor-control/matlab-analysis/blob/master/smr%20to%20mat.s2s

**Other**		

CED 1401 mk2	Cambridge Electronic Design (CED)	N/A
Digitimer NL844 pre-amplifier	Digitimer	SKU: NL844
Digitimer NL820A isolator	Digitimer	SKU: NL820A
Stimulation apparatus Digitimer DS3	Digitimer	SKU: DS3
Microscope	Kaps	SOM 82
Heating bath	VWR	462-8557
Magnetic stirrer	VWR	442-0185
IV apparatus	Harvard Apparatus	Cat# 55-2222
Silver ball electrodes	In-house	N/A
Grounding electrodes	In-house	N/A
Stimulation electrodes	In-house	N/A
Electrode holders	In-house	N/A
Head fixation set	In-house	N/A
Snout stabilizing ring	In-house	N/A
Stabilizing ear rods	In-house	N/A
Heating pad	In-house	N/A
1 mL syringe	VWR	613-4971
5 mL syringe	VWR	613-1997
20 mL syringe	VWR	613-3916
50 mL beaker	VWR	213-0462
Pipettes	Sarstedt	86.1180
Cotton	Apoteket	N/A
Spongostan	Apoteket	257220
Bone wax	AgnTho’s	W31C
IV tube	In-house	N/A
Hair clipper	Aesculap, Inc.	CAT# GT421
Scalpel handle	AgnTho’s	10003–12
Scalpel blade	AgnTho’s	BB510
Tissue forceps	AgnTho’s	11021-15
Vein catheter	BD	SKU: 427421
Dissection scissors	AgnTho’s	14117-14
Surgical fine forceps	AgnTho’s	11231-30
Vascular scissors	AgnTho’s	15000-04
Microsurgical forceps (tip size 0.005 × 0.25mm)	AgnTho’s	11252-00
Catheter tweezers	AgnTho’s	11282-11
Forceps	AgnTho’s	13008-12
Surgical suture	AgnTho’s	14777
Wound retracting hooks	In-house	N/A
Fixed scalpel	Fisher Scientific	10719611
Bone Rongeur forceps (cup size 0.5 mm)	AgnTho’s	16221-14
Bone wax scalpel	In-house	N/A
Dental drill: model 317N	Silfradent	N/A

## Materials and equipment

**Table:** IP anesthesia

Reagent	Final concentration	Amount
Ketaminol vet	100 mg/mL	1 mL
Rompun vet	20 mg/mL	0.3 mL
**Total**	**77 mg/mL/4.6 mg/mL**	**1.3 mL**

***Note:*** Can be mixed ahead of time and stored in −4°C for 7 days.

**Table:** IV anesthesia

Reagent	Final concentration	Amount
Ketaminol vet	100mg/mL	2 mL
Rompun vet	20 mg/mL	0.5 mL
Ringer-acetate	N/A	11 mL
Glucose	50 mg/mL	11 mL
**Total**	**8 mg/mL/0.4 mg/mL**	**24.5 mL**

***Note:*** Can be mixed ahead of time and stored in −4°C for 7 days.

**Table:** Agar

Reagent	Final concentration	Amount
Agar-Agar	3%	6 g
Saline	0.9%	20 mL
**Total**	**N/A**	**20 mL**

***Note:*** Dilute 6 g of agar in 20 mL of saline solution (0.9%) in a glass beaker, Add a stirring magnet and put on a spinning plate to heat and mix until the agar is completely dissolved. Keep on the plate to ensure it does not set before it is used (step 3).

## Step-by-step method details

### Craniotomy and electrode placement

**Timing: 1 h**

This step goes through how to perform the craniotomies, as well as doing all the preparation and set up to start the recording.

**Figure 6:**
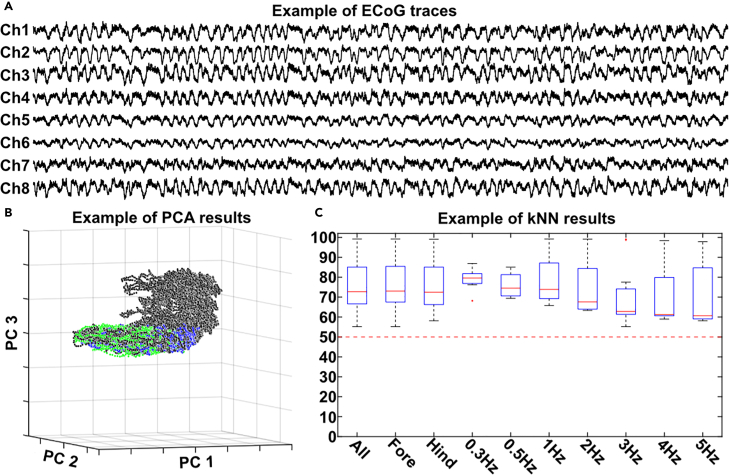
Expected outcomes (A) The 8 simultaneous ECoG traces, one minute pictured. (B) An example of how the activity distribution might look in the PC space. (C) An example of the kNN results. The y-axis show the resulting kNN accuracy (%). Dashed red line indactes chance level. (B and C adapted from the corresponding paper Mellbin et al. 2024).

1. Accessing the skull.
  a. Using a scalpel, make an incision on top of the head, from just above the eyes to just before the neck.
  b. Using a blunt, fixed, scalpel, scrape of any connective tissue on the skull, freeing the bone all the way to temporal crest.
    ***Note:*** The skull needs to be clear of soft tissues from a few millimeters above the coronal suture to a few millimeters below the lambdoid suture.
  c. Pull the skin clear from the planned craniotomy areas by using wound retracting hooks.
  d. Clean any blood from the surface of the skull and make sure it is dry.
2. Performing the craniotomy.
  a. Using the dental drill, remove most of the bone in the areas for the craniotomy (Figure 5C).
    i. Drill for 10 s then pause.
    ii. Use a cotton swab drenched in 0.9% NaCl to cool off the bone surface.
    iii. After soaking the bone surface, dry it of carefully with a dry cotton swab.
      ***Note:*** Keep the drilling area dry to ensure the drill can properly remove bone. Troubleshooting 2.
      **CRITICAL:** Do not apply pressure while drilling as that can damage the underlying cortical tissue. Troubleshooting 3.
      **CRITICAL:** Do not drill for more than 10 s between breaks, to avoid tissue damage in the cortex from vibrations and heat.
      **CRITICAL:** Do not drill immediately above the transversal sinus and be careful when drilling close to the lambdoid suture, as rupturing the sinuses in this area will lead to heavy bleeding.
  b. Once only a thin layer of bone is left, use bone Rongeur forceps to cut away the remaining bone and make the opening the desired size.
    **CRITICAL:** Keep the bone Rongeur forceps horizontal to the brain surface and take care not to damage the underlying cortical tissue.
  c. If there is bleeding emanating from the cut bone edges in the craniotomies, put strips of Spongostan on the bleeding edges to keep the cortical tissue free of blood.
  d. Put a swab of cotton soaked in 38°C NaCl solution over the exposed cortical tissue.
    **CRITICAL:** It is important to cover exposed areas of the brain to prevent drying and to protect the exposed cortical tissue during the building of the cotton reinforced pool.
  e. Put small strips of Spongostan at the edges of the resected skin around the skull to keep blood from pooling in the craniotomies.
3. Cotton reinforced agar pool building.
  a. Use scissors to create a small opening in the skin in the neck of the rat and implant the ground electrodes in the neck musculature.
    **CRITICAL:** Implant the ground electrodes before building the agar pool, as doing it after can disrupt the pool and cause leakage.
  b. Make cotton strips between 1–3 centimeters.
  c. Dip the cotton strips in the heated agar solution (40°C) and wipe of excess agar on a piece of paper or a single use glove.
    **CRITICAL:** Make sure the agar is not too hot when applied to the tissue, to avoid tissue damage.
  d. Put the agar-soaked cotton on the skin surrounding the skull.
    **CRITICAL:** Ensure there are no gaps between strips or between strips and tissue were leakage might occur. Make the walls of the pools high enough to hold the paraffin.
  e. Let the agar cool and set.
  f. Remove the cotton on top of the exposed cortical tissue and fill the pool with 38°C paraffin oil.
    **CRITICAL:** Control that the pool is not leaking. The cortex must be covered with the paraffin oil to prevent the tissue drying out during recording.
4. Recording set-up.
  a. Affix the recording electrodes to electrode stands around the rat.
  b. Carefully put the recording electrodes (silver ball silver electrodes Φ250 μm) one by one on the surface of the designated cortical surface (Figure 5B).
    **CRITICAL:** Make sure the electrodes are not touching, to prevent disruptions to the recording of individual areas.
    ***Note:*** This protocol has been performed on rats ranging between ∼300–700 grams. Using rats weighing below 300 grams is not recommended due to the cranium being too small to easily do the four craniotomies and fitting the 8 recording electrodes.
  c. Plug in the recording electrodes to the pre-amplifier.
    **CRITICAL:** Ensure that the correct electrode is plugged in to the correct socket, so that the ECoG traces correspond to the correct area.
  d. Plug in the ground electrodes to the preamplifier.
  e. Control that the ECoG traces look as expected (Figure 6A). Troubleshooting 4.
  f. Insert the stimulation electrodes at the base of a finger on the wanted paws.
    **CRITICAL:** Make sure that the correct stimulation output is connected to the stimulation electrodes.

### Recording and post-processing

**Timing: 0.5–8 h**

This step covers the recording process as well as the post-processing of the collected data.

5. Record the ECoG traces with the desired stimulation protocol.

***Note:*** Make sure to occasionally check that the pool is not leaking and that blood is not pooling around the recording electrodes to ensure recording quality.

**CRITICAL:** Make sure to keep track of the level of anesthesia and the rats vital status. Troubleshooting 5.

6. Ending the experiment.
  a. With the ECoG trace still running, inject pentobarbital (0.5 mL) through the vein catheter.
  b. Check that breathing and brain activity stops.
  c. Remove all equipment and dispose of the rat according to local guidelines.
7. Post-processing of ECoG data.
  a. If using Spike2 and MATLAB, run all Spike2 files through the “smr to mat.s2s” script in Spike2.
  b. In MATLAB, load the files that will be structs (structure arrays) containing vectors for each ECoG trace and timestamps for stimulation pulses for each stimulation electrode.
  c. Put all the ECoG data together in a single matrix.
  d. Prepare a separate matrix containing the stimulation impulses.
  e. Two more matrixes should be made with times for start and stop points for each stimulation period.
  f. Using the time points for each stimulation impulse, remove any stimulation artifacts with linear interpolation between the two time-steps just before and after the stimulation impulse.
  g. Smooth the raw ECoG data with a Savitzky-Golay filter with a 20 ms window size.

***Note:*** MATLAB requires an activation license to use. Other programs such as Python could potentially be used but will not be a part of this protocol.

### PCA

**Timing: 0.5–8 h**

This step covers performing the principal component analysis in MATLAB. Timing will depend on the amount of data and computer specifications.

8. Preparing the data.
  a. Taking the filtered ECoG data, transpose it if needed so that each column corresponds to an ECoG trace and each row corresponds to a single time step.
  b. Z-score (zero-center) the data using the MATLAB “*zscore*” function.
  c. Compute the PC vector on the entire dataset using MATLAB “*pca*” function. Troubleshooting 6.
  d. The PC vectors can then be divided into spontaneous or stimulated activity using the stimulation time stamps and plotted against each other.

### kNN

**Timing: 1 day–3 weeks**

This step goes through the k-nearest neighbor analysis (kNN) that is used to quantify the separation between spontaneous and stimulated activity. Timing will depend on the amount of data, the number of experiments and the computer specifications.

9. Preparing the data with classifications for the kNN analysis.
  a. Decide what ECoG trace should be used if not all based on which areas are of interest in the analysis.
  b. If any area is excluded from the analysis, remove the corresponding ECoG trace from the data set.
  c. Using the raw, unfiltered, data to ensure the filtering does not skew the results, transpose the data if needed as in step 8a, and perform the PCA.
  d. Normalize all time series of PC coefficients to between 0 and 1, to make sure no PC is given higher or lower weight in the kNN.
  e. Divide the data set into each individual spontaneous and stimulated period.
  f. Stimulated periods should only include the data from 5 ms to 190 ms after each stimulation impulse for that specific stimulation period.
    ***Note:*** This window length is to avoid overlap for the 5 Hz stimulation frequency, while also being longer than any evoked response, which lasts between 20–50 ms. Removing the first 5 milliseconds post impulse is to avoid contamination of the analysis by any shock artifacts.
    ***Note:*** The window size would have to be reduced if stimulation frequencies above 5 Hz are to be analyzed using this method.
  g. To make sure the kNN result is not skewed by difference in group size, check that the difference in the amount of data points for the spontaneous and stimulated activity is not larger than 10%.
  h. Decide what PC vectors should be used if not all and if the stimulated period should be compared to a mix of the spontaneous period before and after the stimulated period, or to the two spontaneous periods separately.
  i. Create a matrix for each group to compare (stimulated and 1 or 2 spontaneous), containing data from the desired ECoG traces.
  j. Add a column in the matrixes that contains a classification label for the data, different labels for each group compared.
  k. Using MATLABs classification learner toolbox, perform kNN on the stimulated period and corresponding spontaneous periods, using N=5 nearest neighbors and five-fold cross validation.
  l. Redo the kNN a 100 times to get a mean decoding accuracy.
  m. If there is a need to only use part of a period, randomize what part is used and redo this every 10^th^ iteration of the classification analysis, to ensure that the reported accuracy is not a result of a specific subset of activity.
  n. Redo steps g–j for each period of stimulated activity.

## Expected outcomes

Successfully completing this protocol results in 8 traces of simultaneous ECoG (Figure 6A) which enables creating graphical representations of how the activity distribution of the cortex changes between spontaneous and stimulated activity using PCA (Figure 6B), as well as allowing for a quantification of this difference using kNN (Figure 6C). By removing different areas or PCs from the data used in the kNN analysis it can also help identify how widespread and high dimensional these changes are, by investigating if separation between different types of activity relies on specific areas or is limited to certain dimensions. The protocol also covers recording ECoG data from several areas which is closely related to the EEG recordings in humans.

This protocol can as such be used to study how an interconnected cortical network could work under different conditions, as demonstrated in Mellbin et al. 2024.

## Quantification and statistical analysis

Data from experiments where the recorded data may be corrupted by damage of the cortical tissue, pool leaking, electrode interference, stimulation not working properly, should be excluded. To evaluate the significance of the kNN decoding accuracy, a random permutation test can be used, where the kNN is reperformed but with shuffled labels, giving the chance level of decoding accuracy.

The kNN results for different groups of stimulation types (e.g. stimulation frequency, stimulation location) should be presented as the median decoding accuracy for all the stimulation periods in the group. To compare results for different groups Wilcoxon rank-sum test can be used.

## Limitations

As this protocol uses separate silver ball electrodes mounted on stands, with separate craniotomies for different areas, spatial constraints will limit the recording potential to 8 simultaneous ECoG traces. The protocol also necessitates the use of anesthesia during the recording, which could limit the study as only sleeping patterns of ECoG activity will be available to record.

Anesthesia levels deeper than necessary might affect the ECoG activity. Additionally, if the room where the recording is done is not reasonably free from electrical interference this could lower the quality of the recorded data.

## Troubleshooting

### Problem 1

The femoral vein is damaged during preparation or when preparing the opening for the catheter (before you begin, step 9).

### Potential solution

If the damage is minor, try tying the vein off below the opening and inserting the catheter. If the damage is major, tie off the vein to stop the bleeding and restart the procedure on the other side.

### Problem 2

A bleeding starts from the bone during the drilling of the skull (step 2).

### Potential solution

Put pressure on the spot using a cotton swab for 10–20 s to see if this stops the bleeding. If not, apply bone wax to stop it.

### Problem 3

The cortex gets damaged from the drilling or bone Rongeur forceps (step 2).

### Potential solution

Terminate the experiment.

### Problem 4

The ECoG traces are low amplitude, have a lot of noise, have large square waves or move outside the expected voltage range for example (step 4).

### Potential solution

For low amplitude, make sure the level of anesthesia is not too deep. Double-check that no filters are used in Spike2 and that all settings on the pre-amplifier are correct. For noise, ensure no unnecessary electrical equipment is on in the recording area. Ensure the recording electrodes are not touching. Check that the pool is not leaking leaving the cortex dry and that no blood is pooling around the recording electrodes. For bigger issues, no trace at all or big disturbances, try deblocking the pre-amplifier. Also double check that the electrodes are touching the brain surface, that the electrodes are not broken and that all equipment is correctly connected.

### Problem 5

The level of anesthesia is insufficient to inhibit the withdrawal reflex. (step 5).

### Potential solution

Administer a bolus injection of 0.2–0.5 mL of anesthesia and increase the drip slightly. If it is not remediated, double check that the catheter is not blocked and has not fallen out. If the catheter no longer works the experiment should be terminated as there is no good way to keep the animal anesthetized.

### Problem 6

The computer starts lagging or stops responding while running the PCA or kNN (step 8).

### Potential solution

Make sure there are not other programs that are also using up a lot of computing power. If this still happens when running only MATLAB a switch to a computer with more computing power is needed.

## Resource availability

### Lead contact

Further information and requests for resources and reagents should be directed to and will be fulfilled by the lead contact, Astrid Mellbin, astrid.mellbin@med.lu.se.

### Technical contact

Technical questions on executing this protocol should be directed to and will be answered by the technical contact, Astrid Mellbin, astrid.mellbin@med.lu.se.

### Materials availability

This study did not generate any new unique reagents.

### Data and code availability

• Original ECoG data reported in this paper will be shared by the lead contact upon request.
• This paper does not report original code.
• Any additional information required to reanalyze the data reported in this paper is available from the lead contact upon request.

## Acknowledgments

This work was funded by the Swedish Research Council VR, project no. 2019-01623.

## Author contributions

Conceptualization, F.B.; methodology, A.M., U.R., and F.B.; software, A.M. and U.R.; validation, A.M.; formal analysis, A.M.; investigation, A.M. and F.B.; resources, F.B.; writing – original draft, A.M.; writing – review and editing, A.M., U.R., and F.B.; visualization, A.M.; supervision, F.B.; funding acquisition, F.B.

## Declaration of interests

The authors declare no competing interests.

## References

[1] Mellbin A., Rongala U., Jörntell H., Bengtsson F. (2024). ECoG activity distribution patterns detects global cortical responses following weak tactile inputs. iScience.

[2] Nietz A.K., Popa L.S., Streng M.L., Carter R.E., Kodandaramaiah S.B., Ebner T.J. (2022). Wide-Field Calcium Imaging of Neuronal Network Dynamics in vivo. Biology.

[3] Findling C., Hubert F., Laboratory I.B., Acerbi L., Benson B., Benson J., Birman D., Bonacchi N., Carandini M., Catarino J.A. (2023). Brain-wide representations of prior information in mouse decision-making. bioRxiv.

[4] Wahlbom A., Enander J.M.D., Jörntell H. (2021). Widespread Decoding of Tactile Input Patterns Among Thalamic Neurons. Front. Syst. Neurosci..

[5] Maloney K.J., Cape E.G., Gotman J., Jones B.E. (1997). High-frequency gamma electroencephalogram activity in association with sleep-wake states and spontaneous behaviors in the rat. Neuroscience.

